# Unaccusativity and Grammatical Aspect: A Cross-Modal Lexical Priming Study

**DOI:** 10.1007/s10936-026-10221-4

**Published:** 2026-06-01

**Authors:** Nermina Čordalija, Roelien Bastiaanse, Toivo Glatz, Seçkin Arslan, Srđan Popov

**Affiliations:** 1 Faculty of Philosophy, University of Sarajevo, Sarajevo, Bosnia and Herzegovina; 2 Institute of Public Health, Charité - Universitätsmedizin Berlin, Freie Universität Berlin and Humboldt-Universität Zu Berlin, Berlin, Germany; 3 Laboratoire Bases, Corpus, Langage (BCL), Université Côte d’Azur, CNRS, Nice, France; 4 Centre for Language and Cognition Groningen (CLCG), University of Groningen, Groningen, The Netherlands

**Keywords:** Aspect, Imperfective, Perfective, Unaccusative, Unergative, Lexical priming, Filler-gap dependencies, Bosnian/Croatian/Serbian (BCS)

## Abstract

Unergative verbs assign the agent theta role to subjects whereas unaccusative verbs occur with theme subjects. Theory suggests that, unlike subjects of unergative verbs, theme subjects of unaccusative verbs are merged in the post-verbal internal argument position and moved to the pre-verbal external argument position. In a cross-modal lexical priming experiment, we tracked (re‑)activation patterns of the subject in sentences with imperfective and perfective unergative and unaccusative verbs in Bosnian/Croatian/Serbian (BCS). We investigated the interplay between unaccusativity and verbal aspect. Our findings are that the subject of perfective unaccusative verbs is (re‑)activated post-verbally, at the gap position, whilst this is not the case for unergative verbs and imperfective unaccusative verbs.

## Introduction

Unaccusativity and grammatical aspect (hereafter *aspect*) are two linguistic concepts that have been extensively studied in many languages (e.g., Unaccusativity: Perlumutter, 1978; Hoekstra, 1984; Burzio, 1986; Grimshaw, 1990; Pullum, 1991; Aspect: Comrie, 1976; Brecht, 1985; Gasparov, 1990; Smith, 1991), but the relationship between the two has hardly been addressed experimentally. In the current paper, we describe a psycholinguistic experiment on the interplay between unaccusativity and aspect in Bosnian/Croatian/Serbian (BCS).

### Unergativity and Unaccusativity

Argument structure is part of a verb’s lexical entry that provides information about the number of arguments a verb takes, their categorical features, and their theta-roles with respect to the verb (Babby, 2009; Belletti & Rizzi, 1988; di Sciullo & Williams, 1987; Marantz, 1984; Radford, 2009; Williams, 1981). Verbs differ with regards to the number and type of arguments they take (Ackema, 2015; Babby, 2009; Koeneman & Zeijlstra, 2017). In this study, we focus on sentences with intransitive verbs, that is, verbs that have a single argument in the subject position. According to the Unaccusative Hypothesis there are two classes of intransitive verbs: unergative and unaccusative verbs (Burzio, 1986; Perlmutter, 1978; Pullum, 1991).

The syntactic description of unaccusative verbs is best formulated when discussed in the context of the usual dichotomy of *unergativity* vs. *unaccusativity*. Unergative verbs assign the agent role to the subject of the sentence, whereas unaccusative verbs, which lack an agent, have a theme argument in the subject position (Florian Radford, 2009; Schäfer, 2009). The Unaccusative Hypothesis (Perlmutter, 1978) argues that the single argument of unaccusative verbs (the theme) originates as the direct object, in the internal argument position. Similarly, Burzio (1986) posited that the sole argument of unergative verbs is an external argument while the one of unaccusative verbs is an internal argument. Such a theoretical framework entails that unaccusative verbs do not have a constituent in the external argument position (Burzio, 1986; Grimshaw, 1990; Hoekstra, 1984; Perlmutter, 1978; Perlmutter & Postal, 1984). Consequently, sentences with unergative and unaccusative verbs are derived differently, as can be seen in (1–2).

1. Unergative [_TP_[_DP_the girl] [_VP_ran]].
  **Figure:**
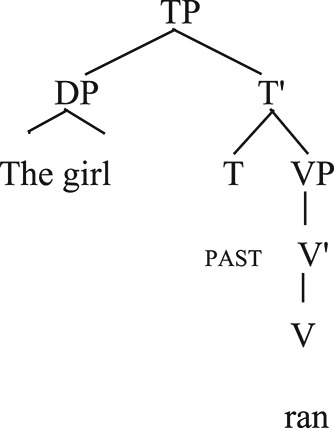
2. Unaccusative [_TP_[_DP_the girl_i_] [_VP_fell t_i_]].
  **Figure:**
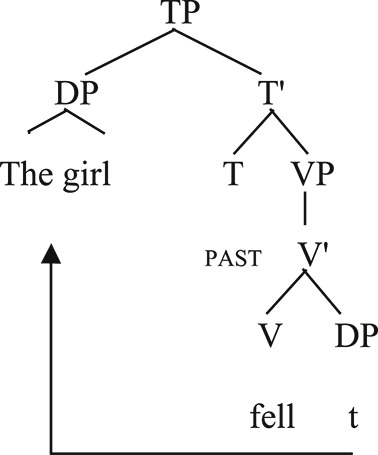

The subject of the unergative verb in (1) is generated in the pre-verbal position, where it superficially occurs. In (2) the theme subject of the unaccusative verb is initially merged in the internal argument position and then moved to the pre-verbal external argument position. The reason, according to Burzio's Generalization (1986), is that only verbs that can assign a theta role to its subject can assign the accusative case to their object. Since the object cannot receive the accusative case from the verb, it moves to the subject position and receives the nominative case accordingly.

The derivation above is described in the context of the Government and Binding framework (Chomsky, 1981). Other theoretical frameworks also posit the difference between unaccusative and unergative verbs. In Minimalism (Chomsky, 1995), sentences with unergative verbs involve only External Merge operations whilst sentences with unaccusative verbs include External and Internal Merge (Re-merge) operations. We provide a syntactic analysis of unaccusativity in the context of Government and Binding (originally proposed in Chomsky, 1981) following other studies (e.g., Friedmann et al., 2008; Thompson, 2003).

To experimentally investigate the above-mentioned differences between the theta roles of the subjects of unergative and unaccusative verbs, we studied the (re‑)activation patterns of the subject in sentences with those two types of verbs. Since BCS is a Slavic language with a binary opposition between imperfective and perfective aspect, the interplay between aspect and unaccusativity is of interest as well. For that reason, in the next section, we address formal properties of BCS aspect.

### Aspect in Bosnian/Croatian/Serbian (BCS)

In BCS, aspectual information is expressed by lexical aspect (telic vs. atelic) and grammatical aspect (perfective vs. imperfective in 3 and 4 below). Lexical aspect relates to the inherent features of the verb (de Swart, 2012; Filip, 2019), such as telicity. Atelic events do not have an inherent end-point but an arbitrary one (*plivati*: ‘to swim’), whilst telic events have a natural endpoint (*pojesti jabuku*: ‘eat an apple’) and denote actions tending towards a goal (Garey, 1957; Padučeva, 2009; Smith, 1991, 1997, 2013).

Grammatical aspect as defined by Comrie (1976, 1985) is a grammaticalised expression of the internal temporal constituency of an event. In other words, aspect provides information about temporal contours of an event in the sentence (Flecken et al., 2015). Perfective grammatical aspect looks at the event as a whole from the outside, without recognizing different phases that make up the event (Comrie, 1976; Gasparov, 1990; Smith, 1991, 1997, 2013). Imperfective grammatical aspect views the situation internally and makes stages of the situation, as well as its participants, temporal and spatial properties semantically visible (Comrie, 1976; Filip, 1999; Madden & Zwaan, 2003; Smith, 1991, 1997, 2013) without specifying its endpoints (Smith, 1991, 1997, 2013).

In BCS, most verbs are inherently imperfective, deriving the perfective form by prefixation as in (3) (Riđanović, 2012) or by changing the suffix in the stem of the imperfective as in (4) (Čirgić et al., 2010; Silić & Pranjković, 2007).

(3) * pisati*_IPFV_ – *napisati*_PFV_: ‘to write’
  Sekretarice su pisale pisma cijelu noć.
  Secretaries AUX_PRS_ write_PTCP.IPFV_ letters all night.
  ‘The secretaries were writing letters all night.’
  Sekretarice su napisale pisma za jedan sat.
  Secretaries AUX_PRS_ write_PTCP.PFV_ letters in one hour.
  ‘The secretaries wrote the letters in one hour.’
(4) kucati_IPFV_—kucnuti_PFV_: ‘to knock’
  Poštar je kucao na vrata dugo vremena.
  Postman AUX_PRS_ knock_PTCP.IPFV_ on door long time.
  ‘The postman was knocking on the door for a long time.’
  Poštar je kucnuo na vrata jednom.
  Postman AUX_PRS_ knock_PTCP.PFV_ on door once.
  ‘The postman knocked on the door once.’

Even though generally, this does not need to be the case, perfective verbs used in the present study encoded telicity. The next section discusses the interplay between grammatical aspect and unaccusativity. More specifically, we explain the inextricable link between telicity, perfective aspect and unaccusativity.

### Unaccusativity, Time Reference, and Aspect in BCS and Cross-linguistically

Perlmutter (1978) argues that intransitive telic verbs tend to show unaccusative behaviour, while intransitive atelic verbs tend to be unergative. In addition to aspectual preferences, Torrence and Hyams (2005) show that English-speaking children prefer the past tense with telic verbs and the present tense with atelic verbs. This suggests that argument structure, telicity (i.e., lexical aspect) and time reference are connected. These concepts of aspect and time reference are combined in the Aspect Assignment Model (AAM) designed by Bastiaanse and Platonov (2015). Relying on the idea that intransitive telic verbs tend to be unaccusative and that intransitive atelic verbs tend to be unergative (Perlmutter, 1978), they make the following two assumptions (a reduced version of AAM, p. 148): i) unergative verbs prefer imperfective aspect, so perfective aspect is a conflict, and ii) unaccusative verbs prefer perfective aspect, imperfective aspect is a conflict.

Furthermore, based on the criteria typically used to test unaccusativity, Aljović (2000) argues that imperfective unaccusative verb forms in BCS do not show typical unaccusative behaviour and that their arguments do not show the behaviour typical of internal arguments. Aljović (2000) discusses several tests, including impersonal passive constructions. These constructions are normally not available to unaccusative verbs, whereas impersonal passive constructions with unergative verbs (such as *walk* in 5) are felicitous.

(5) Po ovom ćilimu je mnogo hodano.
  On this carpet AUX a lot walked_PTCP_

‘There was a lot of walking on this carpet.’

Perfective unaccusative verb forms yield ungrammatical examples when used in an impersonal passive construction. On the contrary, imperfective unaccusative verb forms can be used in the impersonal passive constructions shown in (6).

(6) Na ovu pistu je često slijetano.^1^
  On this runaway AUX often landed_PTCP.IPFV_

‘This runway was often landed on.’

Aljović's ideas and tests tie in nicely with the AAM, that is, unaccusative verbs are interpreted as being perfective and the combination of unaccusativity and imperfective aspect is conflicting. However, in the Government and Binding theoretical framework (Chomsky, 1981) unaccusative structures are derived by movement of the internal argument to the external argument position and it is unclear whether the loss of unaccusative properties (i.e., the failure to pass standard unaccusativity tests) in imperfective aspect entails that theme subjects of imperfective unaccusative verb forms are base-generated in the external argument position. This would reject the Universal Theta Assignment Hypothesis (UTAH; Baker, 1988) that assumes that all agents are base-generated in the external argument position and all themes are base-generated in the internal argument position.

There are no studies that experimentally explore the relationship between unaccusativity and perfectivity as described in Aljović (2000). However, a similar theoretical challenge is encountered with psychological verbs (hereafter *psych-*verbs) that also have the theme in the subject position and, in their case, the experiencer in the object position (*The climate change worries Giovanni*; Belletti & Rizzi, 1988) and with the so-called ‘mixed verbs’ (*The diamond sparkled*; Koring et al., 2012). Since psych-verbs have a theme argument in subject position too, there were different proposals about how to account for the fact that a theme, a theta-role typical of the internal argument position, occurs in the external argument position.

Belletti and Rizzi (1988) apply the unaccusative analysis to psych-verbs. Non-movement approaches suggest that the subject of psych-verbs is base-generated in the external argument position (Arrad, 1998; Pesetsky, 1995)*.* However, the non-movement approaches do not treat the argument in the subject position as a theme, but rather describe it in terms of other thematic roles (e.g., *causer* in Pesetsky, 1995).

In the context of the present study, mixed verbs are more relevant as mixed verbs also have a theme argument in the subject position, just like unaccusatives and psych-verbs, but unlike psych-verbs, mixed verbs are intransitive, as are unaccusative verbs. Reinhart (2000) argues that mixed verbs have a thematic structure of unaccusative verbs but syntactic structure of unergative verbs.

Similarly, Koring et al. (2012) explain that even though mixed verbs occur with the theme argument in the subject position, they do not show unaccusative behaviour (e.g., they select the auxiliary *hebben* ‘to have’ in Dutch, just like unergatives do and unlike unaccusatives that select the auxiliary *zijn* ‘to be’). In an experiment using the visual world paradigm, Koring and Mak (2010) as well as Koring et al. (2012) show that mixed verbs (*sparkle*) show the same across-sentence activation patterns of the theme subject as unergative verbs with the agent subject and that the subject activation patterns in sentences with unaccusative verbs and mixed verbs differ, even though they both have theme subjects.

Koring et al. (2012) conclude that the reason why several studies find differences in processing unergative and unaccusative verbs lies in the difference between internal and external arguments and not necessarily in the agent—theme difference. Mixed-verbs and unergative verbs assign different theta roles to their subjects (theme and agent, respectively), however, both their subjects are base-generated in the external argument position. What follows from Koring and Mak (2010) as well as Koring et al. (2012) is that unaccusative verbs have the same thematic structure as mixed verbs, but not the same syntactic structure. The subjects of unaccusative verbs are base-generated in the internal argument position. Experimental studies by Koring and Mak (2010) as well as Koring et al. (2012) do not support the UTAH: theme subjects of mixed verbs are base-generated as external and not as internal arguments.

We hypothesise that imperfective unaccusative verb forms show the same type of behaviour that mixed verbs show. They have theme subjects (just like perfective unaccusatives) but they do not show unaccusative behaviour in standard unaccusativity tests (they show unergative-like behaviour). To test our hypothesis, we investigated the activation patterns of subjects in sentences with imperfective and perfective unaccusative verb forms. For this, we used the *Cross-Modal Lexical Priming* technique.

### Cross-Modal Lexical Priming

Cross-modal lexical priming (CMLP; Swinney et al., 1979) is an online behavioural task that provides insight into activation patterns during ongoing sentence comprehension. The method has frequently been used in the past to investigate syntactic processing (e.g., De Goede, 2006; Friedmann et al., 2008; Love, 2007; Love & Swinney, 1996; Nicol & Swinney, 1989; Roberts et al., 2007; Swinney et al., 1989). The first two elements of the name, ‘cross-modal’, imply that the task involves different modalities, auditory and visual: sentences are presented auditorily and at one or more points in the sentence, words, referred to as ‘probes’, are visually presented. Probes can be related to the critical word, unrelated to it or they can be a non-word. The participant is asked to perform a secondary task, that is, to indicate by a button press whether the probe is an existing word or not. The idea is that when the meaning of the critical word is active, a button press to a semantically related word is faster than to a semantically unrelated word. In other words, there is ‘lexical priming’ at the position where the meaning of the critical word is active. Lexical priming assumes facilitation of the response latencies after a semantically related prime. Observe the following example from Marinis (2010).

(7) John saw the peacock_i_ to which the penguin gave the nice birthday present* t*_*i*_ in the garden

The example in (7) is a case of a filler-gap dependency where the indirect object from the subordinate clause (the filler) has been moved to the direct object position in the main clause leaving a gap at the position where it was originally merged. The filler (‘antecedent’ in linguistic terminology) and the gap (‘trace’ in linguistic terminology) establish a dependency relation which means that the filler is stored in the short term memory until the point of the gap where the meaning of the filler is reactivated (Marinis, 2010). The reactivated meaning of the filler will serve as a prime for the semantically related probe at the gap. Thus, the response time is expected to be faster than for an unrelated probe that will not be primed.

Probes are usually placed immediately after the first occurrence of the filler, at the gap and some time after the gap. The last probe position is included based on findings that the filler re-activation can have different temporal courses. In an experiment on *wh*-movement, re-activation of the filler was shown to occur at the gap without delay (Nicol & Swinney, 1989). Osterhout and Swinney (1993), however, found that re-activation in passives occurs not immediately at the gap but 1000 ms after the verb.

### Unaccusativity in English

Friedmann and colleagues (2008) tested subject activation patterns during online processing of unergative and unaccusative verbs in English in a cross-modal lexical priming experiment. In order to test whether the subject of unaccusative verbs is initially merged in the internal argument position and then undergoes movement, Friedmann et al. (2008) observed (re-)activation patterns of the head-word of the subject in three positions: after the head-word of the subject NP, after the unaccusative or unergative verb (the original position of the theme subject of unaccusative verbs) and 750 ms after the verb. The results show that the meaning of the head-word of the subject is activated at the subject position. This is the only time activation can be observed for unergative verbs. However, in sentences with unaccusative verbs, the meaning of the head-noun of the subject is re-activated 750 ms after the verb (but not directly after the verb). This was attested by faster response times to words that were semantically related to the subject (*garden* in 8) than to unrelated words (*butter* in 8) 750 ms after the verb (position 3 in 8) [example from Friedmann et al., 2008].

(8) The rose **[1]** outside the entryway to the expensive home finally bloomed **[2]** after the owners **[3]** were already completely desperate. (probe: *garden/butter*)

The authors claim that in the case of unaccusative verbs, the lexical semantic features of the theme (that was moved from the post-verbal to the subject position) are re-activated with a delay of 750 ms after the verb. Since the subject of unergative verbs never originated post-verbally, no re-activation of the meaning of the subject occurs in post-verbal positions.

Our study was similar in design to Friedmann et al.’s (2008) study. However, there are a few important differences. Friedmann et al. (2008) included two types of unaccusatives: those that have a transitive counterpart such as *to break* and those that do not, such as *to fall*. We did not include verbs with alternating transitivity as in BCS, alternating unaccusatives are morphologically more complex than English ones and occur with the clitic *se* that is not present in non-alternating unaccusatives. Instead, we focus on the interaction between aspect and unaccusativity that was not investigated in Friedmann et al. (2008).

### The Present Study

We performed a cross-modal lexical priming experiment to investigate the online processing of unaccusative verbs in BCS. We focus on the post-verbal position, the position where the subject noun is supposed to be base-generated in the sentences with unaccusative verbs. On the basis of the theories discussed above, two hypotheses were formulated. The first hypothesis is based on the findings of Friedmann et al. (2008) and the second one on the theory that imperfective unaccusative verbs behave like unergative verbs.

#### Hypothesis 1

In BCS constructions with perfective unaccusative verbs, the meaning of the moved subject will be re-activated at the post-verbal gap position, which will result in faster response times to semantically related than to semantically unrelated probes for perfective unaccusative verbs, but not for unergative verbs.

#### Hypothesis 2

BCS constructions with imperfective unaccusative verb forms will have the same activation patterns of the subject as unergative verbs: no re-activation of the meaning of the subject at the post-verbal positions.

## Method

### Participants

Participants in our study were 120 undergraduate students (90 females, age range 18 – 25 years, mean age 20.1 years) at the University of Sarajevo, Bosnia and Herzegovina. Participants were L1 speakers of the Bosnian variant of BCS, with the exception of two participants who were native speakers of Montenegrin. The exclusion criteria entailed normal or corrected-to-normal vision and hearing and no history of previous language or reading disorder or neurological injury. Participants either received course credit or 5 euros for participation. With the information sheet, participants were informed of the duration of the experiment and the procedure. They were told that they could withdraw from the experiment at any time. All the data obtained from participants were anonymised by assigning a numerical code to each participant. The study was approved by the Research Ethics Committee (CETO) of the Faculty of Arts, University of Groningen, The Netherlands.

### Materials and Design

In the CMLP study, experimental and filler sentences comprised three probe positions: after the head of the subject NP (probe position 1), immediately after the verb or at the position of the gap in case of unaccusative sentences (probe position 2) and, following Friedmann et al. (2008), 750 ms after the verb (probe position 3). This is illustrated in (9).

(9) Glumica **[1]** u crvenoj suknji ukrašenoj zlatnim nitima i ružičastim.
  Actress_NOM_ in red skirt_DAT_ embroidered golden threads_INST_ and pink.
  cvjetovima je pala **[2]** niz stepenice dok je.
  flowers_INST_ AUX_PRS_ fall_PTCP.PFV_ down stairs while AUX_PRS_.
  voditelj dodjele Oskara **[3]** čitao njenu biografiju.
  host ceremony Oscar_GEN_ read_PTCP.IPFV_ her biography.

‘An actress** [1]** in a red skirt embroidered with golden threads and pink flowers.

fell **[2]**down the stairs while the host of the Oscars **[3]** was reading her biography.’

One of the ways to ensure that participants perform at an optimal level in a CMLP experiment is a proper design of the probes. Designing the probes involved several steps. The first step was to distribute a questionnaire to 15 native speakers of BCS, the Bosnian variant, who were instructed to write the closest semantic associate of the nouns (the subject NPs of experimental and filler sentences) they were presented with. The most frequently mentioned semantic associate was chosen as the related probe.

For every related probe, three unrelated probes were selected that matched the related probe in the number of letters and word frequency. A single unrelated probe was selected based on the results of the lexical decision task in E-prime (E-prime 2.0) that we performed among 20 native speakers of BCS. The lexical decision task included related probes and three potential unrelated probes for each subject NP (240 items) plus the same number of non-words respecting the phonotactic restrictions in BCS which yielded 480 items in total. Therefore, related and unrelated probes were also matched on baseline response time. Examples of related and unrelated probes are given in sentences (10–13).

The experimental sentences comprised 15 unaccusative verbs and 15 unergative in BCS. Unergative verbs served as a control condition**,** since their subjects do not move from the internal to the external argument position, and no interaction with aspect was expected. All unaccusative verbs passed two unaccusativity tests to confirm their unaccusative properties: left-branch extraction and impersonal passive constructions (see Aljović, 2000). Each unergative and unaccusative verb was used twice – in imperfective and perfective aspect. The experiment comprised four conditions illustrated in (10–13).^2^

Unergative perfective:

(10) Poštar sa pismom u jednoj ruci i paketom u drugoj *je.*
  Postman_NOM_ with letter in one hand and package in another AUX_PRS_.
  *pokucao* na vrata nekoliko puta ali ga mladić nije.
  knock_PTCP.PFV_on door several times but he_ACC_ boy_NOM_ AUX_PRS.NEG_.
  čuo zbog slušalica na ušima.
  hear_PTCP.PFV_ because headphones_GEN_ on ears.

‘A postman with a letter in one hand and a package in another *knocked* on the door several times but the young man did not hear him because of the headphones.

Related probe: pismo (*letter*)

Unrelated probe: motor (*engine*)

unergative imperfective:

(11) Takmičar iz najpoznatijeg kluba za plivanje u čitavoj Evropi *je.*
  Contestant_NOM_ from most famous club for swimming in whole Europe AUX_PRS_.
  *plivao* do cilja sa lakoćom uprkos nedavnoj povredi na olimpijskim.
  swim_PTCP.IPFV_ to finish line with ease despite recent injury at Olympic.
  igrama.
  games.

‘A contestant from the most famous swimming club in all of *Europe was swimming* to the finish line with ease despite a recent injury at the Olympics.’

Related probe: takmičenje (*competition*)

Unrelated probe: provalnik (*burgular*)

Unaccusative perfective:

(12) Glumica u crvenoj suknji ukrašenoj zlatnim nitima i ružičastim.
  Actress_NOM_ in red skirt_DAT_ embroidered golden threads_INST_ and pink.
  cvjetovima *je pala* niz stepenice dok je.
  flowers_INST_ AUX_PRS_ fall_PTCP.PFV_ down stairs while AUX_PRS_.
  voditelj dodjele Oskara čitao njenu biografiju.
  host ceremony Oscar_GEN_ read_PTCP.IPFV_ her biography.

‘An actress in a red skirt embroidered with golden threads and pink flowers *fell *down the stairs while the host of the Oscar ceremony was reading her biography.’

Related probe: film (*film*)

Unrelated probe: njiva (*field*)

Unaccusative imperfective:

(13) Vodostaj Drine na granici Bosne i Hercegovine i.
  Water-level_NOM_ Drina_GEN_ on border Bosnia and Herzegovina_GEN_ and.
  Srbije u ponoć *je* *opadao* na radost stanovnika.
  Serbia_GEN_ at midnight AUX_PRS_ subside_PTCP.IPFV_on luck inhabitants_GEN_.
  okolnih mjesta koji su se plašili poplava.
  surrounding areas who AUX_PRS_ PRT fear_PTCP.IPFV_ floods.

‘The water level of the Drina on the border between Bosnia and Herzegovina and Serbia *was subsiding* at midnight luckily for the inhabitants of the surrounding areas who were fearing floods.’

Related probe: rijeka (*river*)

Unrelated probe: sestra (*sister*)

As illustrated in (10–13), each sentence was designed to be structurally similar. The first element was an NP with a postmodifying phrase consisting of 7–9 words. This allowed sufficient time for a decay in activation between the first appearance of the head-word of the subject NP and the gap (in the case of perfective unaccusative verbs). Next came the perfect periphrastic verb form (the contracted present tense auxiliary 'biti' [*be*] + a non-finite participle) expressing past time reference. The periphrastic verb form was followed by an adverbial phrase/clause that allowed placement of the probe position 3 (750 ms after the verb).

Animacy of the subject was difficult to control. Since unergative verbs take agent subjects, naturally, subjects tend to be animate. For unaccusative verbs and their theme subjects, it is the opposite – they favour inanimate subjects but animate subjects are not excluded. Hence, in our experiment, unergative verbs had animate subjects only, whilst sentences with unaccusative verbs were approximately balanced between animate and inanimate subjects. Experimental sentences comprised 120 items.

Filler sentences contained transitive verbs that cannot be used intransitively. Filler sentences had an identical structure as the experimental sentences which is shown in (14). Every verb occurred twice – in imperfective and perfective aspect. In total, there were 40 filler verbs, which yielded 80 filler sentences in total.

(14) Sudija Vrhovnog suda sa brojnim riješenim slučajevima iza sebe.
  Judge_NOM_ Supreme Court_GEN_ with numerous solved cases behind him.
  je kaznio osuđene još strožije jer je procijenio.
  AUX_PRS_ punish_PTCP.PFV_ felons more strictly because AUX_PRS_ estimate_PTCP.PFV_.
  da ne pokazuju kajanje za učinjeno djelo.
  that NEG show remorse for past actions.

‘The Supreme Court judge with numerous resolved cases imposed a greater sentence on the felons as they failed to show remorse for their past actions.’

Since there were three probe positions in our experiment design, each sentence had to occur three times with two types of probes: related and unrelated. Using the Latin Square design, the three probe positions and two probe types were equally distributed across six lists with 80 filler sentences in every list. Probes for filler sentences were the non-words. Probes for experimental sentences were all words, either related or unrelated to the subject NP.

### Procedure

The experiment was coded and presented via the Psychology software tool, E-prime (E-prime 2.0). During the experiment, participants sat in front of a computer. The sentences were presented auditorily. Participants wore headphones and were instructed to carefully attend to the sentences. Probes appeared centrally on the monitor for 500 ms and the participants were asked to perform the lexical decision task as quickly and accurately as possible via button press on the computer keyboard. The experiment had one session. Response times were automatically recorded by the computer.

### Data Analysis

We used R 4.5.2 (R Core Team, 2020) and the *lme4* package (Bates et al., 2015) to fit three linear mixed effects regression models (one per probe position) to test whether the dependent variable log-transformed response time varies as a function of the independent variables Probe Type (related and unrelated probes), Verb Type (unergative and unaccusative verbs), and Aspect (perfective and imperfective verbs). The fixed-effects structure used dummy coding for the three-way interaction of these independent variables, whereas the random-effects structure contained random intercepts for subjects and items. Based on this model, post-hoc pairwise comparisons of Probe Type within the four conditions (unergative perfective, unergative imperfective, unaccusative perfective, unaccusative imperfective) were conducted using estimated marginal means (*emmeans* package; Lenth, 2022). All post-hoc results are reported using *p*‑values adjusted for multiple comparisons (Bonferroni method for family-wise error correction).

Following Friedmann et al. (2008), all data points with a response time above 2000 ms as well as incorrect responses were discarded before analysis (0.3% of all trials). The exact number of analysed trials per probe position is reported below.

## Results

**Fig. 1:**
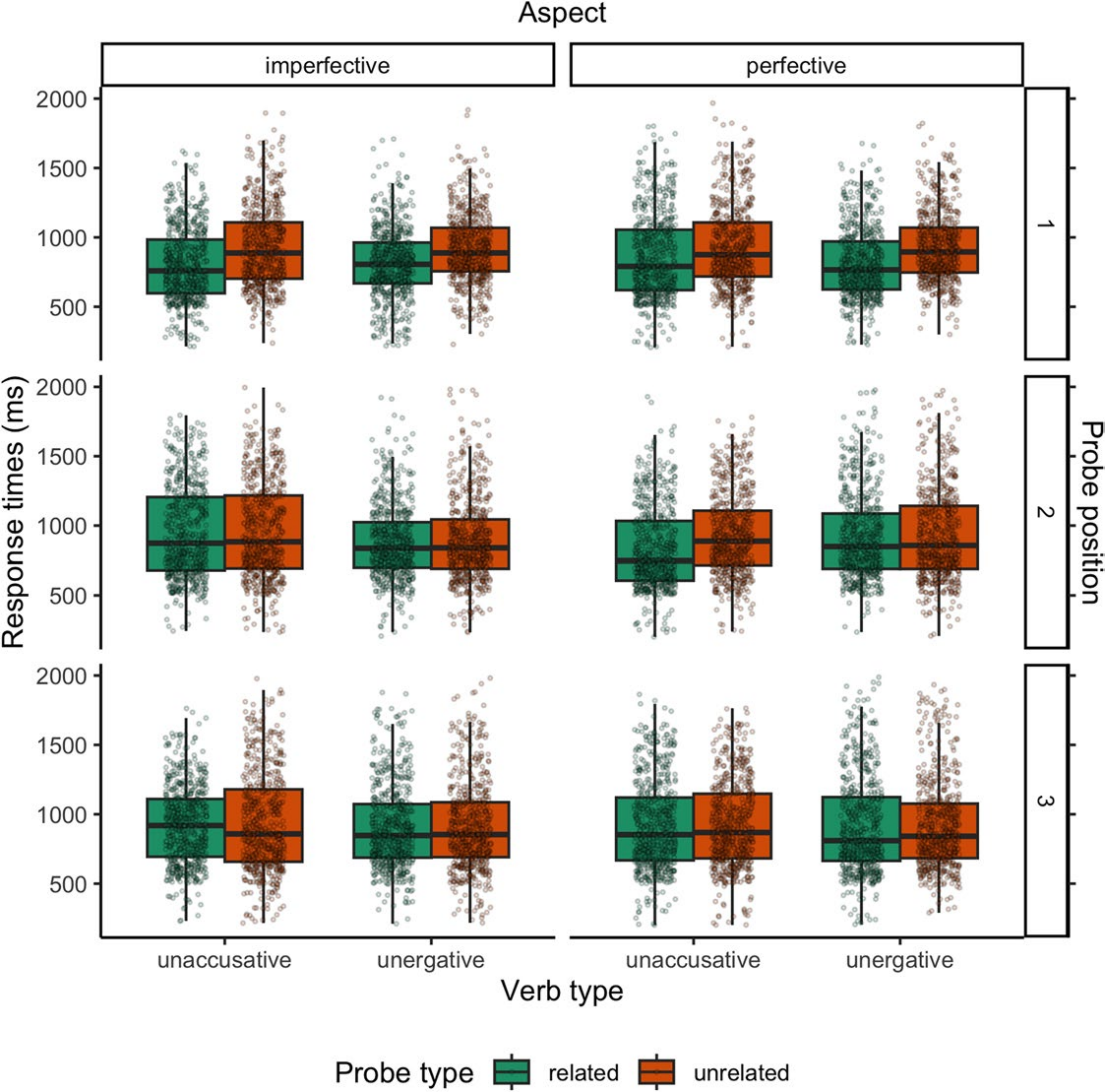
Boxplots of participants’ response times across conditions in milliseconds. Probe position indicates [1] immediately after the head-word of the subject NP; [2] immediately after the verb; and [3] 750 ms after the verb

To observe the subject activation patterns in the four conditions, response times for related probes and response times for unrelated probes were compared in positions 1 (after the head-word of the subject NP), 2 (after the verb) and 3 (750 ms after the verb) in each individual condition to track if lexical priming occurs.^3^ Raw response times across conditions and probe positions with overlaid boxplots are shown in Fig. 1. Median response times and inter quartile range per probe position and probe type across verb types and aspect conditions are shown in Table 1. The coefficients, t-values and p-values from the linear mixed effects regression models per probe position are given in Table 2.

**Table 1:** Median response times and inter quartile range of the participants’ responses across conditions in milliseconds

	Unergatives				Unaccusatives			
	Perfective		Imperfective		Perfective		Imperfective	
Probe position	Related	Unrelated	Related	Unrelated	Related	Unrelated	Related	Unrelated
[1]	766 *(625, 970)*	895 *(747, 1070)*	806 *(669, 962)*	888 *(756, 1069)*	791 *(620, 1055)*	876 *(718, 1107)*	759 *(598, 984)*	888 *(703, 1108)*
[2]	849 *(692, 1086)*	857 *(691, 1142)*	837 *(700, 1024)*	*840* *(692, 1044)*	748 *(606, 1033)*	888 *(716, 1107)*	873 *(680, 1206)*	884 *(694, 1217)*
[3]	810 *(664, 1124)*	842 *(684, 1077)*	847 *(688, 1074)*	854 *(691, 1086)*	853 *(668, 1120)*	868 *(682, 1148)*	919 *(694, 1110)*	858 *(658, 1180)*

Probe position indicates [1] immediately after the head-word of the subject NP; [2] immediately after the verb; and [3] 750 ms after the verb

**Table 2:** Estimates from the mixed-effects regression models per probe position

	[1]				[2]				[3]			
	*β*	*SE*	*t*	*p*	*β*	*SE*	*t*	*p*	*β*	*SE*	*t*	*p*
Intercept	6.63	0.02	327.48	< 0.001	6.80	0.02	291.77	< 0.001	6.78	0.02	283.06	< 0.001
Probe Type (Unrelated)	0.15	0.02	9.80	< 0.001	0.01	0.02	0.42	0.672	-0.01	0.02	-0.51	0.613
Verb Type (Unergative)	0.02	0.02	0.90	0.367	-0.06	0.02	-3.76	< 0.001	-0.02	0.02	-0.98	0.328
Aspect (Perfective)	0.06	0.02	3.54	< 0.001	-0.13	0.02	-8.32	< 0.001	-0.02	0.02	-0.82	0.416
Probe Type × Verb Type	-0.02	0.02	-0.83	0.405	0.00	0.02	0.04	0.966	0.01	0.02	0.23	0.820
Probe Type × Aspect	-0.06	0.02	-2.88	0.004	0.11	0.02	5.35	< 0.001	0.01	0.02	0.59	0.558
Verb Type × Aspect	-0.06	0.02	-2.52	0.012	0.16	0.02	7.49	< 0.001	0.01	0.03	0.25	0.803
Probe Type × Verb Type × Aspect	0.08	0.03	2.59	0.010	-0.12	0.03	-3.96	< 0.001	0.01	0.03	0.23	0.816

Coefficients of fixed effects (β) show log-transformed response times

### Probe Position 1

At the first probe position immediately after the head-word of the subject NP, the linear mixed effects regression model was based on 4785 observations (see Table 2 for model summary). Pairwise post-hoc analyses revealed a significant effect of Probe Type within each of the four conditions, with related probes yielding faster response times than unrelated probes throughout (unergative perfective: estimate = -0.15, *t* = -9.73, *p* < 0.001; unergative imperfective: estimate = -0.14, *t* = -8.63, *p* < 0.001; unaccusative perfective: estimate = -0.09, *t* = -5.72, *p* < 0.001; unaccusative imperfective: estimate = -0.15, *t* = -9.79, *p* < 0.001). In other words, lexical priming occurred upon the presentation of related probes in probe position [1] for both verb types and for both types of aspect.

### Probe Position 2

**Fig. 2:**
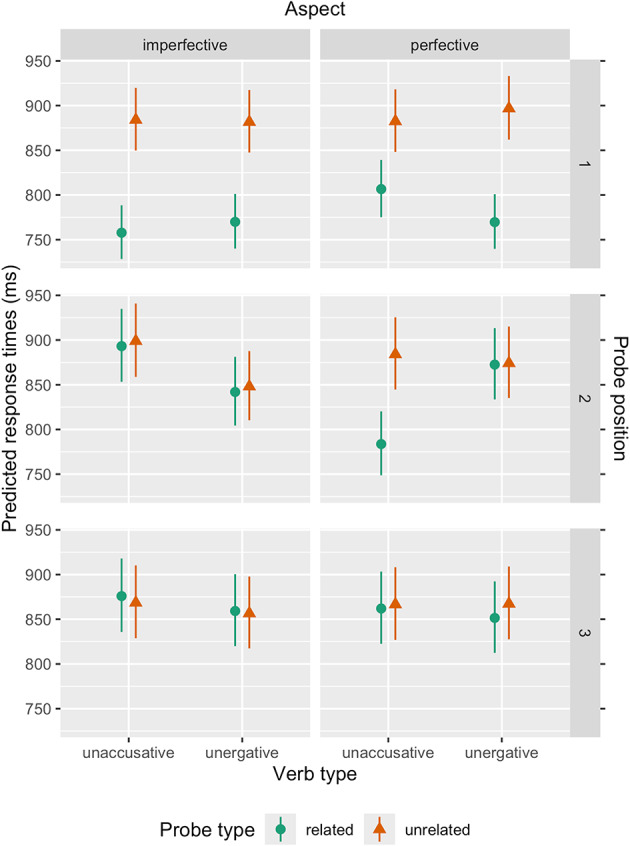
Estimated average response times based on the three-way interaction of Verb Type × Probe Type × Aspect at each probe position. Error bars indicate 95% confidence intervals

At the second probe position immediately after the verb, the model was based on 4789 observations (see Table 2 for model summary). Pairwise post-hoc analyses revealed a significant effect of Probe Type within the unaccusative perfective condition, but none in any of the other three conditions (unergative perfective: estimate = -0.00, *t* = -0.12, *p* = 1.000; unergative imperfective: estimate = -0.01, *t* = -0.48, *p* = 1.000; unaccusative perfective: estimate = -0.12, *t* = -7.99, *p* < 0.001; unaccusative imperfective: estimate = -0.01, *t* = -0.42, *p* = 1.000). Thus, facilitation in response times upon the presentation of the related probe only occurred when unaccusative verbs were combined with perfective aspect (Fig. 2).

### Probe Position 3

At the third probe position 750 ms after the verb, the model was based on 4784 observations (see Table 2 for model summary). Pairwise post-hoc analyses found no effect of Probe Type in any of the conditions (unergative perfective: estimate = -0.02, t = -1.11, p = 1.000; unergative imperfective: estimate = 0.00, t = 0.18, p = 1.000; unaccusative perfective: estimate = -0.01, t = -0.32, p = 1.000; unaccusative imperfective: estimate = 0.01, t = 0.61, p = 1.000). Therefore, no lexical priming occurred.

## Discussion

The CMLP results suggest that in probe position [1], related probes were responded to faster compared to unrelated probes across all four experimental conditions: perfective unergative verbs, imperfective unergative verbs, perfective unaccusative and imperfective unaccusative verbs. This pattern is expected, as probe position [1] immediately follows the presentation of the head of the subject NP, serving as a baseline control for lexical priming. The consistent facilitation indicates that all subject NPs were successfully activated upon initial presentation.

The results further suggest that in probe position [2] it is only for perfective unaccusative verbs in BCS that the meaning of the moved subject is re-activated at the postverbal gap position, leading to faster response times for semantically related probes compared to unrelated probes. No such effect was observed for perfective unergative verbs, imperfective unergative verbs, or imperfective unaccusative verbs. In other words, the results show that aspect effects are restricted to unaccusatives and do not occur with unergatives. Activation patterns for unergative verbs remained consistent across both imperfective and perfective aspect. This highlights that the facilitation observed in response times is specific to the interaction of unaccusativity and perfective aspect, and not a general property of all intransitive verbs. Since aspect effects were neither expected nor found for unergative verbs, for practical reasons, hereafter, we refer to unergative verbs without specifying their aspectual marking.

In probe position [3], no priming effect was observed in any condition. This absence of facilitation is consistent with the finding that subject re-activation occurs specifically at the postverbal gap position (probe position [2]**)** for perfective unaccusative verbs, and not in the other conditions.

We will relate these findings to the hypotheses formulated in the Introduction. This will be followed by a section in which we compare our BCS findings to the results of Friedmann et al. (2008) for English unaccusatives.

### Unergativity Versus Unaccusativity

The data from our CMLP experiment support the first hypothesis that in BCS constructions with perfective unaccusative verbs, the meaning of the moved subject is reconstructed at the gap position. The evidence for this is faster response time to semantically related words than semantically unrelated words at the gap position for perfective unaccusative verbs. This priming effect, that is, the difference in response times to related as opposed to unrelated probes in probe position 2, was not found for unergative verbs implying that the meaning of the subject is not active after the verb in sentences with unergative verbs.

Therefore, the results of the CMLP experiment support the theoretical claims we made in the Introduction. Sentences with unergative verbs underlyingly have an Agent-Verb word order, whereas sentences with perfective unaccusative verb forms have an underlyingly Verb-Theme word order. Perfective unaccusative verb forms initially merge their theme argument in the internal argument position and then, in the course of the derivation, this argument is moved to the pre-verbal subject position, which means that unaccusativity is syntactically encoded. This is a complex syntactic operation. Interestingly, the results are consistent with aphasiological findings: agrammatic speakers have more difficulties producing the more complex (sentences with) unaccusative verbs compared to other intransitive verbs (Bastiaanse & Platonov, 2015; Bastiaanse & Van Zonneveld, 2004, 2005; Lee & Thompson, 2004; Thompson, 2003).

Importantly, as discussed in the Introduction, we should not consider the unaccusative verb forms as a homogeneous group. Aspect plays a decisive role in the way a sentence with an unaccusative verb is derived.

### Interplay Between Unaccusativity and Aspect

The behavioural results support the second hypothesis that imperfective unaccusative verb forms have the same subject activation patterns as unergative verbs. In both cases, there is no reactivation of the meaning of the subject noun after the verb in the absence of lexical priming. On the contrary, the behavioural results showed that in sentences with perfective unaccusative verb forms, the meaning of the subject noun (the theme) is re-activated at the gap position. Therefore, the CMLP results suggest a strong link between perfective aspect and unaccusativity – the unaccusative properties are lost when the verb is marked for the imperfective aspect which has the following manifestations: 1) imperfective unaccusative verb forms do not pass standard unaccusativity tests; 2) the meaning of the subject of imperfective unaccusative verbs forms is not active immediately after the verb or 750 ms after the verb, suggesting that the subject of imperfective unaccusative verb forms must be base-generated in the external argument position.

However, the priming effect in probe position 2 in sentences with perfective unaccusative verbs does not necessarily entail that the meaning of the subject is re-activated. It only shows that the meaning of the subject is active at the gap position. Nevertheless, since the distance from the head of the subject NP and the verb was 7–9 words and since the meaning of the head of the subject NP is no longer active in the sentences with unergative verbs and imperfective unaccusative verbs, we assume that the meaning of the head-noun of the subject in sentences with perfective unaccusative verbs forms was first deactivated and at the gap, it was re-activated which is why priming occurred in case of the related probes. The fact that no such re-activation of the meaning of the subject was found in sentences with imperfective unaccusative verbs calls forth certain theoretical implications that are discussed in the next section.

### The Issue of Imperfectivity

The CMLP data demonstrate the expected interplay between unaccusativity and the category of aspect. Based on Aljović (2000) and Bastiaanse and Platonov (2015), we hypothesised that unaccusativity is strongly linked to perfective aspect and that only the perfective unaccusative verb form shows unaccusative behaviour. Our results confirm this. The subjects of sentences with imperfective unaccusative verb forms are not base-generated in the internal argument position and, as expected, have similar activation patterns as subjects of unergative verbs: the meaning of the subject is not re-activated at the post-verbal position(s).

The lack of re-activation of the subject at the gap position in sentences with imperfective unaccusative verb forms entails that theme subjects of imperfective unaccusatives are base-generated in the external argument position. In other words, while the perfective unaccusative verbs show canonical unaccusative behavior, sentences with imperfective unaccusative verbs show the same pattern of derivation as sentences with unergative verbs. For theoretical linguistics, our data present the same challenge to the UTAH as the so-called mixed verbs – intransitive verbs with theme subjects (*The diamond sparkled*; Koring et al., 2012).

Koring and Mak (2010) and Koring et al. (2012) experimentally showed that mixed verbs (*sparkle, stink, shine, itch*) show the same across-sentence activation patterns of the theme subject as unergative verbs with the agent subject and that the subject activation patterns in sentences with unaccusative verbs and mixed verbs differ, even though they both have theme subjects. Koring et al. (2012) conclude that the theme subjects of mixed verbs are base-generated in the external argument position.

We argue for the same non-movement account of sentences with imperfective unaccusative verb forms. Imperfective unaccusative verbs forms and unergative verbs assign different theta roles to their subjects (theme and agent, respectively), however, their subjects are base-generated in the external argument position. Perfective unaccusative verb forms have the same thematic structure as imperfective unaccusative verb forms (theme arguments), but not the same syntactic derivation. The subjects of perfective unaccusative verb forms are base-generated in the internal argument position where they were re-activated as evidenced by the presence of priming effects.Therefore, imperfective unaccusative verb forms in BCS show the same type of mismatch between thematic structure and syntactic structure (theme base-generated in the external argument position) as do mixed verbs in Koring and Mak (2010) and Koring et al. (2012). Consequently, our experimental data are in line with these studies in that they do not support the UTAH: whilst theme subjects of sentences with perfective unaccusative verbs are base-generated as internal arguments, theme subjects of imperfective unaccusative verbs are base-generated as external arguments.

We would like to address one last point regarding our findings. In our stimuli, unergative verbs were combined with animate subjects only, whereas unaccusative verbs had an equal number of animate and inanimate subjects. Therefore, we cannot completely exclude the possibility that subject reactivation might be dependent on the subject being inanimate. Such dependence would explain the absence of subject reactivation in (im)perfective unergative verbs and its presence in perfective unaccusatives. However, it cannot account for the presence of subject reactivation in perfective unaccusatives and the lack of reactivation in imperfective unaccusatives. Both unaccusative groups had the same number of animate and inanimate subjects (approximately half-half). Therefore, based on our findings, we believe that subject animacy does not play a role in subject reactivation. Still, the relationship between argument animacy and priming is something that can be addressed in future research.

### English and BCS Unaccusatives Compared

Friedmann et al. (2008) found re-activation of the meaning of the subject in sentences with unaccusative verbs. However, this re-activation occurred 750 ms after the gap. At this position, we did not find a difference in response times between related and unrelated probes in sentences with perfective unaccusative verbs. Rather, we found the re-activation of the meaning of the subject directly at the gap position. This difference may be attributed to differences between past tense in English and BCS. In English, most of the past forms can also function as an adjective (‘the *closed* door’; ‘the *dried* fruit’). Even though this is surely not the preferred reading in the sentences that were used by Friedmann et al. (2008), when a word is perceived, all possible meanings are activated; only later, the parser homes in on the meaning relevant for the given context (Frisson, 2009; Frisson & Pickering, 1999; Pickering & Frisson, 2001; Swinney, 1979).

In our BCS experimental sentences with perfective unaccusative verbs, such an ambiguity did not exist as the adjectival reading of the perfective unaccusative verb in the perfect periphrastic verb form with auxiliary *je* is not possible. This explains why in BCS re-activation of the filler occurred without a delay. This cross-linguistic difference between English and BCS aside, our study successfully replicated the findings of Friedmann and colleagues (2008): the subjects of (perfective) unaccusative verbs are base-generated in the internal argument position.

## Conclusion

The psycholinguistic experiment reported here is, to our knowledge, the first experimental study on unaccusativity and its interplay with verbal aspect in any language. The study provides novel insights into the processing of sentences with unergative verbs, perfective unaccusative verb forms, and imperfective unaccusative verb forms. Crucially, the results demonstrate an interplay between unaccusativity and perfective aspect: perfective unaccusatives show clear subject reactivation at the postverbal position, whereas imperfective unaccusatives pattern together with unergatives and show no such effect. The results also provide cross-linguistic evidence that the diagnostic properties of unaccusativity identified in languages such as English extend to BCS, but with aspect-related nuances.

## Data Availability

The datasets generated during the current study are available from the corresponding author on reasonable request.

## Experimental Sentences by Verb Type and the Related and Unrelated Probes that Appeared with them

Probes are written in brackets in this order: related probe/unrelated probe. Each sentence contains a verb in the perfective and the imperfective form written in this order and italicised. The head-noun of the subject of the sentence is underlined.

### Sentences with Unaccusative Verbs

1. Glumica u crvenoj suknji ukrašenoj zlatnim nitima i ružičastim cvjetovima *je pala/padala* niz stepenice dok je voditelj Oskara čitao njenu biografiju. **(film/njiva).**
  ‘An actress in a red skirt decorated with golden threads and pink flowers *fell/was falling down* the stairs while the Oscar host was reading her biography.’ **(film/field)**
2. Čistačica zadužena za pranje prozora na devetom spratu Parlamenta *je pala/padala* sa skele dok su građani u nevjerici posmatrali ovu strašnu nesreću. **(metla/slovo).**
  ‘A cleaning lady who was in charge of washing windows on the ninth floor of the Parliament building *fell/was falling* from the scaffolding while the citizens watched this terrible accident in disbelief.’ **(broom/letter)**
3. Pjevačica u raskošnoj haljini dizajniranoj za Dan državnosti odjednom/polako *je nestala/nestajala* izvan dohvata reflektora koji su obasijavali čitavu pozornicu. **(mikrofon/graditelj).**
  ‘A singer in a gorgeous dress designed for the Statehood Day *disappeared/was disappearing* suddenly/slowly out of the reach of the spotlights that illuminated the entire stage.’ **(microphone/builder)**
4. Pedijatar omiljen među djecom zbog svoje vedrine i optimizma *je nestao/nestajao* iza zavjese na pozornici nakon što je dobio aplauz za izvrsnu predstavu/dok su mu djeca aplaudirala za izvrsnu predstavu. **(dijete/općina).**
  ‘A paediatrician loved by children for his cheerfulness and optimism *disappeared/was disappearing* behind the curtains on the stage after/while being applauded for an excellent performance.’ **(child/municipality)**
5. Trener najtrofejnijeg odbojkaškog kluba za djevojke i mladiće *je propao/propadao* na ličnom i profesionalnom planu zbog ovisnosti o kockanju. **(sport/sapun).**
  ‘The coach of the most trophy-winning club for girls and boys *deteriorated/was deteriorating* on a personal and professional level due to his gambling addiction.’ **(sport/soap)**
6. Astrolog sa mnogobrojnim klijentima iz zemlje i inostranstva *je propao/propadao* zbog javnih upozorenja koja su razotkrila njegove brojne prevare. **(zvijezda/trudnoća).**
  ‘An astrologer with numerous clients from the country and abroad *went out of business/was going out of business* due to public warnings that exposed his numerous scams.’ **(star/pregnancy)**
7. Spasilac na jednoj od najvećih brazilskih plaža na trenutak *je iščeznuo/iščezavao* pod velikim talasima u pokušaju da spasi što više ljudi nakon cunamija. **(nesreća/tvrđava).**
  ‘A lifeguard at one of the largest Brazilian beaches *disappeared/was disappearing* (for a moment) under the high waves in an attempt to rescue as many people as possible after the tsunami.’ **(accident/fortress)**
8. Ronilac sa mnogo iskustva u posmatranju bijelih ajkula *je iščeznuo/iščezavao* pod snažnim talasima dok se borio da sačuva kameru i snimke sa dna okeana. **(kisik/pepeo).**
  ‘A diver with a lot of experience in observing white sharks *disappeared/was disappearing* under forceful waves as he struggled to save the camera and footage from the bottom of the ocean.’ **(oxygen/ash)**
9. Automehaničar sa izrazitim talentom sa muziku i poeziju *je umro/umirao* duhom u dekadenciji kapitalizma nadajući se da će dobiti bar jednu priliku da pokaže svoj pravi talenat. **(auto/nebo).**
  ‘A car mechanic with a distinct talent for music and poetry *died/was dying* spiritually when faced with the decadence of capitalism hoping to get at least one chance to show his true talent.’ **(car/sky)**
10. Pjesnik poznat po nekim od najljepših stihova današnjice iznutra *je umro/umurao* pred banalnošću malograđanskog postojanja koje je najviše mrzio. **(poezija/planina).**
  ‘A poet known for some of the most beautiful verses of today *died/was dying* spiritually when faced with the banality of petty bourgeois existence that he detested.’ **(poetry/mountain)**
11. Režiser sa mnogo nagrada sa svjetskih festivala sa zakašnjenjem *je stigao/stizao* u pozorište dok su ga glumci čekali. **(film/kora).**
  ‘A director with numerous awards from world festivals *arrived/was arriving* at the theatre late while the actors were waiting for him.’ **(film/bark)**
12. Vajar iz Meksika bez mnogo iskustva na međunarodnim izložbama trčeći *je stigao/stizao* na otvaranje svoje prve strane izložbe. **(skulptura/radijator).**
  ‘A sculptor from Mexico without much experience in international exhibitions *arrived/was arriving* hurriedly to the opening of his first international exhibition.’ **(sculpture/radiator)**
13. Hokejaš iz kadetskog tima sa mnogo talenta ali malo iskustva *je odrastao/odrastao* u pouzdanog igrača koji je mogao igrati na različitim pozicijama za vrijeme utakmice. **(led/konj).**
  ‘A hockey player from the cadet team with a lot of talent but little experience *grew/was growing* into a reliable player who could play in different positions during the game.’ **(ice/horse)**
14. Džudista iz juniorske selekcije reprezentacije Sjedinjenih Američkih Država *je odrastao/odrastao* u prvaka koji je osvajao medalje na takmičenjima širom svijeta. **(pojas/jelka).**
  ‘A judoka from the junior selection of the United States national team *grew/was growing* up to be a champion that won medals at competitions around the world.’ **(belt/Christmas tree)**
15. Vodostaj Drine na granici Bosne i Hercegovine i Srbije u ponoć *je opao/opadao* na radost stanovnika okolnih mjesta koji su se plašili poplava. **(rijeka/sestra).**
  ‘The water level of the Drina on the border between Bosnia and Herzegovina and Serbia *dropped/was dropping* at midnight luckily for the inhabitants of the surrounding areas who were fearing floods.’ **(river/sister)**
16. Plima u jednom od zaljeva na jugu Norveške *je opala/opadala* kako je polako prolazila vedra noć obasjana punim mjesecom. **(more/jaje).**
  ‘The tide in one of the bays in the south of Norway *dropped/was dropping* as a clear moonlit night slowly passed.’ **(sea/egg)**
17. Aceton u jednoj od najskupljih kozmetičkih radnji u centru grada *je ispario/isparavao* iako je stajao zatvoren na polici vitrine za šminku. **(lak/čip).**
  ‘Acetone in one of the most expensive beauty stores in the city center *evaporated/was evaporating* although it stood closed on the shelf of the make-up display case.’ **(polish/chip)**
18. Parfem kupljen prošle godine kao poklon za rođendan *je ispario/isparavao* stojeći na polici jer ga nikada nije imala priliku dati prijateljici. **(miris/ćilim).**
  ‘A perfume bought last year as a birthday present *evaporated/was evaporating* on the shelf as she never had a chance to give it to her friend.’ **(scent/carpet)**
19. Grana sa ponekim žutim listom preostalim nakon tmurne jeseni *je pukla/pucala* pod težinom snijega kojeg su donijeli hladni decembarski dani. **(kora/vino).**
  ‘A branch with some yellow leaves that survived the gloomy autumn *broke/was breaking* under the weight of the snow brought by the cold December days.’ **(bark/wine)**
20. Daska postavljena umjesto mostića između obale i splava (polako) *je pukla/pucala dok su ribari* su unosili ogromnu gajbu punu svježe ulovljene ribe. **(stolar/jabuka).**
  ‘A board installed between the shore and the raft as a bridge replacement *cracked*/*was* slowly *cracking* as fishermen brought in a huge crate full of freshly caught fish.’ **(carpenter/apple)**
21. Glas poštara zakopčanog do grla i sa rukama u džepovima *je odjeknuo/odjekivao* pustim ulicama Starog grada jednog sumornog januarskog jutra. **(grlo/vila).**
  ‘The voice of a postman buttoned up to his throat and with his hands in his pockets *resonated/was resonating* through the empty streets of the Old Town one gloomy January morning.’ **(throat/fairy)**
22. Smijeh djece zaigrane u parku tog sunčanog dana *je odjeknuo/odjekivao* našom ulicom upotpunjujući svu ljepotu koju odlazak hladne zime donosi. **(radost/gorivo).**
  ‘The laughter of the children playing in the park that sunny afternoon *resonated/was resonating* through our street accentuating the beauty brought by the end of the winter.’ **(joy/fuel)**
23. Rat sa brojnim žrtvama među vojnicima kao i civilima (polako) *je prestao/prestajao* čim je mirovni sporazum konačno potpisan. **(masakr/sijeno).**
  ‘The war with numerous casualties among soldiers as well as civilians *ceased/was* slowly *ceasing* as soon as the peace agreement was signed.’ **(massacre/hay)**
24. Borba između oružanih snaga dvije države oko strateški važnog grada *je prestala/prestajala* zbog intervencije međunarodne zajednice na radost tamošnjeg stanovništva. **(vojnik/struja).**
  ‘The fighting between the armed forces of the two countries over the strategically important city *ceased/was ceasing* due to the intervention of the international community to the joy of the local population.’ **(soldier/current)**
25. Vrt sa raznobojnim cvjetovima tulipana, ruža i orhideja *je postao/postajao* sumoran u predvečerje kada su sunčeve zrake polako nestale. **(trava/banka).**
  ‘A garden with colourful tulips, roses and orchids *became/was becoming* gloomy in the evening as the sun’s rays slowly disappeared.’ **(grass/bank)**
26. Voćnjak od nekoliko hektara zemlje sa mnogo stabala šljive *je postao/postajao* tmuran i siv kada/kako je došla/dolazila jesen i donijela/donosila kišovite dane. **(voće/kosa).**
  ‘An orchard of several acres of land with many plum trees *became/was becoming* gloomy and grey as the autumn and rainy days set in.’ **(fruit/hair)**
27. Ustanak protiv neefikasne i korumpirane osmanke vlasti (postepeno) *je izbio/izbijao* u svim dijelovima carstva nakon što je carski savjet odlučio uvesti nove namete stanovništvu. **(pobuna/makaze).**
  ‘An uprising against the inefficient and corrupted Ottoman government *broke out/was* gradually *breaking out* in all parts of the empire after the imperial council decided to impose new levies on the citizens.’ **(rebellion/scissors)**
28. Sukob na sjevernoj granici Turske i Sirije odjednom (polako) *je izbio/izbijao* a mediji su zataškavali stvarnu priču o sukobu ove dvije zemlje.** (svađa/šnala).**
  ‘The conflict on the northern border between Turkey and Syria *suddenly broke out*/*was* slowly *breaking out* and the media covered up the real story behind the conflict between these two countries.’** (quarrel/hair pin)**
29. Voda iz česme prekrivene hrđom i slojevima buđavog taloga *je kapnula/kapala* nekoliko puta/neprestano ali majstor je brzo našao način da popravi česmu. **(piće/pila).**
  ‘Water from a tap covered in rust and layers of mouldy sediment *dripped* several times/*was dripping* incessantly but the plumber quickly found a way to repair it.’ **(drink/saw)**
30. Krv iz desne nozdrve učenika prvog razreda osnovne škole *je kapnula/kapala* nekoliko puta/dugo vremena na klupu pa je učiteljica iz predostrožnosti pozvala roditelje. **(rana/guma).**
  ‘Blood from the right nostril of a first-grader *dripped/was dripping* on the bench several times/for a long time so the teacher called the student’s parents as a precaution.’ **(wound/tire).**

### Sentences with Unergative Verbs

1. Poštar sa pismom u jednoj ruci i paketom u drugoj *je pokucao/kucao* na vrata nekoliko puta/dugo vremena ali ga mladić nije čuo zbog slušalica na ušima. **(pismo/motor).**
  ‘A postman with a letter in one hand and a package in another *knocked/was knocking* on the door several times/for a long time but the young man did not hear him because he was wearing headphones. **(letter/engine)**
2. Vodoinstalater sa ogromnom crvenom kutijom punom alata *je pokucao/kucao* na staklena vrata kancelarije za sastanke nekoliko puta/dugo vremena ali mu niko nije otvarao. **(cijev/kruna).**
  ‘A plumber with a huge red box of tools *knocked/was knocking* on the glass door of the meeting room several times/for a long time but no one opened.’ **(pipe/crown)**
3. Beba sa rozom vunenom kapom na glavi uplašeno *je dopuzala/puzala* do mekanog ćilima bježeći od klizavog parketa dnevnog boravka u porodičnoj kući. **(plač/blic).**
  ‘A baby with a pink woollen hat shyly *crawled/was crawling* to the soft carpet fleeing from the slippery living room floor in the family house.’**(crying/[camera] flash)**
4. Zmija duga četiri metra i široka pedesetak centimetara *je dopuzala/puzala* do centra bine dok su akrobati izvodili opasne vratolomije na veliko zadovoljstvo publike. **(otrov/tabla).**
  ‘A snake four meters long and about fifty centimetres *wide crawled/was crawling* to the centre of the stage while the acrobats were performing dangerous stunts to the great satisfaction of the audience.’ **(poison/board)**
5. Takmičar iz najpoznatijeg kluba za plivanje u čitavoj Evropi *je doplivao/plivao* do cilja sa lakoćom uprkos nedavnoj povredi na olimpijskim igrama. **(takmičenje/provalnik).**
  ‘A contestant from the most famous swimming club in all of *Europe swam/was swimming* to the finish line with ease despite a recent injury at the Olympics.’ **(competition/burglar)**
6. Pacijentica sa krivom kičmom i isčašenim kukom *je doplivala/plivala* do polovine olimpijskog bazena kao dio svakodnevne vježbe.** (bolnica/sudnica).**
  ‘A patient with a crooked spine and a dislocated hip *swam/was swimming* to the middle of the Olympic pool as part of a daily exercise. **(hospital/courtroom)**
7. Starica jako dobrog zdravlja i sa mnogo vjere u budućnost *je zaplesala/plesala* sa svojim mužem na pedesetoj godišnjici mature što je nagrađenom velikim aplauzom. **(štap/kiša).**
  ‘An old woman in very good health and with a lot of faith in the future *danced/was dancing* with her husband on the fiftieth graduation anniversary, which was rewarded with a big applause.’ **(walking stick/rain)**
8. Preduzetnik sa mnogo iskustva u poslovanju sa inozemnim firmama *je zaplesao/plesao* sa svojom suprugom na otvaranju nove podružnice u Tokiju. **(preduzeće/pepeljara).**
  ‘An entrepreneur with a lot of experience in cooperation with foreign companies *danced/was dancing* with his wife at the opening of a new branch in Tokyo.’ **(enterprise/ashtray)**
9. Atletičar iz siromašne i opasne četvrti glavnog grada Italije *je otrčao/trčao* do svlačionice/po mokroj stazi zbog/nakon strašnog proloma oblaka tog tmurnog subotnjeg popodneva. **(sport/jufka).**
  ‘An athlete from a poor and dangerous neighbourhood of the Italy’s capital *ran/was running* to the locker room/on a wet track due to/after a terrible cloudburst that Saturday afternoon.’ (**sport/pastry**)
10. Konj uzgajan za galopske utrke sa zaprekama u Velikoj Britaniji *je otrčao/trčao* do cilja sa lakoćom i ostvario najbolji rezultat sezone. **(kopito/prsluk).**
  ‘A horse bred for gallop races over obstacles in Great Britain *ran/was running* to the finish line with ease achieving the best result of the season.’ **(hoof/vest)**
11. Vojnik izgubljen u prašumi nakon neuspješne vojne vježbe *je odveslao/veslao* do obale uz pomoć puške nadajući se da će pronaći put do vojnog kampa. (**rat/rog**).
  ‘A soldier lost in the rainforest after an unsuccessful military drill *paddled/was paddling* to the shore with the help of a rifle hoping to find his way to the military camp.’ **(war/horn)**
12. Naučnik zadužen za pronalazak rijetkih i ugroženih vrsta ajkula *je odveslao/veslao* do grebena nakon što je uočio neobično pomjerenje vode koje je moglo uzrokavati prisustvo ajkula. **(teorija/kutlača).**
  ‘A scientist in charge of finding rare and endangered species of sharks *paddled/was paddling* to the reef after noticing unusual movements in water that could have suggested the presence of sharks.’ **(theory/ladle)**
13. Delfin omiljen kako među djecom tako i među odraslim *je zaronio/ronio* u bazen(u) nekoliko sekundi a onda izveo trik koji je nagrađen velikim aplauzom. **(more/gora).**
  ‘A dolphin loved by children and adults *dived/was diving* into the pool for a few seconds and then performed a trick that was rewarded with a big round of applause.’ **(sea/hill)**
14. Biolog zadužen za ispitivanje nivoa kiselosti okeana *je zaronio/ronio* u vodu/vodi sa opremom pokušavajući pronaći prikladan uzorak vode iz dubine okeana. **(biljka/zlato).**
  ‘A biologist in charge of examining the acidity of the ocean *dived/was diving* in(to) the water with equipment trying to find a suitable water sample from the depths of the ocean.’ **(plant/gold)**
15. Leopard iz rezervata za velike mačke u Meksiku *je zarežao/režao* na hijene koje je kiša okrijepila i ohrabrila na lov. **(krzno/račun).**
  ‘A leopard from the wildcat conservation centre in Mexico *growled/was growling* at hyenas energised by rain and ready to hunt. **(fur/receipt)**
16. Vuk iz najvećeg i najpoznatijeg splitskog zološkog vrta *je zarežao/režao* na čuvare kada su ga pokušali nahraniti u namjeri da zaštiti svoje mlade. **(čopor/torta).**
  ‘A wolf from the largest and most famous zoo in Split *growled/was growling* at the guards to protect the offspring when they tried to feed him.’ **(pack/cake)**
17. Domar zaposlen u jedinoj osnovnoj školi u gradu jednom/stalno *je zazviždao/zviždao* u hodniku za vrijeme časa nakon čega je dobio opomenu direktora/prekidajući tok nastave i ometajući učenike. **(popravak/asteroid).**
  ‘A janitor employed in the only elementary school in town *whistled/was whistling* once/constantly in the hallways during lessons after which he was reprimanded by the principal/which disrupted the teaching process and distracted the students.’ **(repair/asteroid)**
18. Stomatolog na privatnoj klinici za dentalnu medicinu jednom/stalno *je zazviždao/zviždao* u kabinetu što se nije svidjelo jednoj pacijentici te ga je prijavila glavnom odboru. **(zub/top).**
  ‘A dentist at a private dental clinic *whistled/was whistling* once/often in the office which one of his patients did not like so she reported him to the main board.’ **(tooth/cannon)**
19. Labrador sa dvije krupne smeđe tačke iznad očiju *je zalajao/lajao* na glasove koji su dolazili iz daljine i činili se prijetećim. **(pas/krš).**
  ‘A Labrador with two large brown spots above his eyes *barked/was barking* at the voices coming from the distance and that seemed threatening.’ **(dog/karst)**
20. Pitbul iz dvorišta sa natpisom “Čuvaj se psa” *je zalajao/lajao* na poštara koji je pokušavao ubaciti poštu kroz otvor na vratima zbog čega mu je ispala torba i mnogobrojne koverte. **(snaga/kolač).**
  ‘A Pit-bull from the yard with the ‘Beware of the dog’ sign *barked/was barking* at the postman who was trying to put the mail through the door opening which caused his bag and numerous envelopes to fall out.’ **(strength/cake)**
21. Fizioterapeut zaposlen na Klinici za ortopediju Kliničkog centra *je požurio/žurio* na sastanak koji je sazvala direktorica u vezi sa najavljenim štrajkom zdravstvenih radnika.** (masaža/peraje).**
  ‘A physiotherapist employed at the Orthopaedic Clinic of the University Medical Centre *rushed/was rushing* to a meeting requested by the general manager to consider the upcoming health workers’ strike.’ **(massage/fins)**
22. Anesteziolog sa Odjela za kardiohirurgiju zeničkog Kliničkog centra *je požurio/žurio* u operacionu salu nakon što mu je javljeno da će operacija početi ranije nego što je zakazano. **(san/zid).**
  ‘An anaesthesiologist from the Department of Cardiac Surgery of the Medical Centre in Zenica *rushed/was rushing* to the operating room after being informed that the surgery would start earlier than scheduled.’ **(sleep/wall)**
23. Šumar zadužen za redovne kontrole nacionalnog parka *je zakoračio/koračao* pažljivo nakon što mu je javljeno da su lovokradice postavile zamke za lisice posvuda u šumi. **(šuma/kralj).**
  **‘**A forester in charge of regular inspections of the national park *stepped/was stepping* carefully after being informed that the poachers had set fox traps everywhere in the forest.’ **(forest/king)**
24. Kustos Hrvatskog prirodnoslovnog muzeja zagrebačkog Gornjeg grada s radošću *je zakoračio/koračao* u novootvorenu salu/novootvorenom salom posebno uređenu za eksponate/posebno uređenom za eksponate. **(muzej/ustav).**
  ‘The curator of the Croatian Museum of Natural History in Zagreb’s Upper Town *stepped/was stepping* happily in the newly opened hall specially decorated for exhibits.’ **(museum/constitution)**
25. Bibliotekar iz dječijeg odjeljenja javne ustanove Biblioteka Sarajeva *je odskijao/skijao* za djecom u sklopu trke za osnovce koju je organizovala ova javna institucija. **(knjiga/hrana).**
  ‘A librarian from the children’s department of the public institution ‘Libraries of Sarajevo’ *skied/was skiing* with children as part of the race for primary school students organised by this public institution.’ **(book/food)**
26. Skijaš sa zavidnom karijerom i mnogim osvojenim prvenstvima *je odskijao/skijao* niz strmu padinu bez trunke straha što je izazvalo oduševljenje kod gledaoca. **(snijeg/rudnik).**
  ‘A skier with an enviable career and many successful championships *skied/was skiing* down the steep slope without a shred of fear which caused delight among the spectators. **(snow/mine)**
27. Papagaj kupljen prošle sedmice u radnji za kućne ljubimce *je izvirio/virio* iza vrata kaveza pogledom tražeći mačku koja ga je ranije prepala. **(ptica/kaput).**
  ‘A parrot bought last week at a pet store *peeked/was peeking* from behind the door looking for the cat that scared him earlier.’ **(bird/coat)**
28. Jelen iz specijalnog zološkog vrta za ugrožene vrste *je izvirio/virio* iza zida plašljivo pa su čuvari odlučili da ga sklone od posjetilaca neko vrijeme. **(rog/hor).**
  ‘A deer from a special zoo for endangered species *peeked/was peeking* from behind the wall timidly so the guards decided to keep it away from visitors for some time.’ **(horn/chorus)**
29. Oftalmolog zaposlen kao savjetnik u optici Loris u Ferhadiji *je prošetao/šetao* nakon posla sa svojim psom kada je sreo jednu pacijenticu. **(naočale/pustinja).**
  ‘An ophthalmologist employed as a consultant in the optical store Loris in Ferhadija *took a walk/was taking a walk* after work with his dog when he met a patient.’ **(glasses/desert)**
30. Novinar poslat kao strani dopisnik u ratnu zonu u Alepu *je prošetao/šetao* razrušenim gradom a onda se uputio u bazu UN-a da napiše izvještaj o posljednjem sukobu. **(vijest/lopata).**
  ‘A journalist sent as a foreign correspondent to the war zone in Aleppo *walked/was walking* through the ruined city and then headed to the UN base to write a report on the latest conflict.’ **(news/shovel)**

## Footnotes

1 Note that although English ‘to land’ can be transitive (“the pilot lands the plane”), its BCS equivalent *slijetjeti/slijetati* is intransitive in this meaning.

2 It was not expected that aspect would interact with unergative verbs, as aspectual marking does not alter their underlying argument structure or processing expectations. Nevertheless, we divided unergative verbs into perfective and imperfective forms to maintain parallelism with the unaccusative conditions.

3 In the paper, we refrain from making comparisons between unergative and unaccusative conditions as unaccusative and unergative sentences could not form minimal pairs and had to involve different prime-target noun combinations.

## Declarations

### Research Involving Human and Animal Participants

The study involved human participants and all procedures were in accordance with the ethical standards of the institutional research committee and with the 1964 Helsinki Declaration and its amendments or comparable ethical standards.
